# Perilla Oil Has Similar Protective Effects of Fish Oil on High-Fat Diet-Induced Nonalcoholic Fatty Liver Disease and Gut Dysbiosis

**DOI:** 10.1155/2016/9462571

**Published:** 2016-03-09

**Authors:** Yu Tian, Hualin Wang, Fahu Yuan, Na Li, Qiang Huang, Lei He, Limei Wang, Zhiguo Liu

**Affiliations:** ^1^School of Biology and Pharmaceutical Engineering, Wuhan Polytechnic University, Wuhan, Hubei 430023, China; ^2^School of Medicine, Jianghan University, Wuhan, Hubei, China; ^3^Department of Blood Transfusion, Tongji Hospital, Tongji Medical College, Huazhong University of Science and Technology, Wuhan, Hubei, China

## Abstract

Nonalcoholic fatty liver disease (NAFLD) is the most prevalent chronic liver disease in developed countries. Recent studies indicated that the modification of gut microbiota plays an important role in the progression from simple steatosis to steatohepatitis. Epidemiological studies have demonstrated consumption of fish oil or perilla oil rich in n-3 polyunsaturated fatty acids (PUFAs) protects against NAFLD. However, the underlying mechanisms remain unclear. In the present study, we adopted 16s rRNA amplicon sequencing technique to investigate the impacts of fish oil and perilla oil on gut microbiomes modification in rats with high-fat diet- (HFD-) induced NAFLD. Both fish oil and perilla oil ameliorated HFD-induced hepatic steatosis and inflammation. In comparison with the low-fat control diet, HFD feeding significantly reduced the relative abundance of Gram-positive bacteria in the gut, which was slightly reversed by either fish oil or perilla oil. Additionally, fish oil and perilla oil consumption abrogated the elevated abundance of* Prevotella* and* Escherichia* in the gut from HFD fed animals. Interestingly, the relative abundance of antiobese* Akkermansia* was remarkably increased only in animals fed fish oil compared with HFD group. In conclusion, compared with fish oil, perilla oil has similar but slightly weaker potency against HFD-induced NAFLD and gut dysbiosis.

## 1. Introduction

Nowadays, overnutrition has become a big problem of human health. Modern gastronomy encourages people to consume sugars, fats, and proteins more than needed, which lead to caloric surplus and a series of metabolic diseases, such as nonalcoholic fatty liver disease (NAFLD) [1]. NAFLD, which characterises a spectrum of hepatic disorders range from simple steatosis to nonalcoholic steatohepatitis (NASH), is the most common cause of chronic liver diseases in developed countries [2]. A “two-hit” hypothesis has been used to explain the pathophysiology of NAFLD: the first hit, fat ectopic accumulation in liver (steatosis), results in liver sensitive to hepatic oxidative stress and inflammation, known as the second hit and leads to NASH [3]. Hyperphagia related excessive caloric consumption, adipose tissue lipolysis activation, and hepatic insulin resistance all contribute to hepatic steatosis, while gut-source endotoxins and proinflammatory cytokines are associated with hepatic inflammation [4].

Recent studies have pointed out that intestinal microbiota plays a crucial role in both “hits” of NAFLD [5]. Intestinal microbiota, including bacteria, archaea, fungi, and viruses, contains almost 150-fold of DNA sequences than host [6]. Intestinal microbiota affects the progress of NAFLD in two major ways. (1) Gut bacteria affect nutrient digestion and absorption and produce secondary products such as medium and short-chain fatty acids [7, 8]. Moreover, intestinal microbiota influences host energy homeostasis; transplanting gut microbiota from obese mice induces body weight elevation in germ-free mice [9]. Another gut microbiota transplantation study illustrates its capability to regulate the expression of hepatic genes for* de novo* lipogenesis [10]. Furthermore, gut microbiota affects the secretion of gut hormones, such as gastric inhibitory peptide, glucagon-like peptide 1, and peptide YY, which contributes to the metabolic modification [11]. (2) Gut microbiota is closely related to host immune system and hepatic inflammation. Gut Gram-negative bacteria are the major source of plasma lipopolysaccharide (LPS). Saturated fatty acids-enriched high-fat diet (HFD) induced endotoxemia leads to obesity and hepatic insulin resistance and partly contributes to the death of gut Gram-negative bacteria such as* Bacteroides *[12, 13]. The HFD also reduces the population of bifidobacteria in intestinum, thereby reducing mucosal barrier and increasing intestinal permeability for translocation of gut LPS into plasma [12, 14, 15]. Besides, LPS and gut bacteria can activate hepatic stellate cells and Kupffer cells to induce liver fibrosis, a process that Toll-like receptors (TLRs) such as TLR5 and TLR9 may be involved [16–18].

N-3 polyunsaturated fatty acids (PUFAs) are believed to have benefits against NAFLD [19]. n-3 PUFAs may attenuate the progress of NAFLD via either regulating lipid metabolism or alleviating hepatic inflammation; both are associated with the function of gut microbiota [20]. Intestinal microbiota has been shown to contribute to the effects of palm oil induced hepatic steatosis [21]. However, the influence of n-3 PUFAs on gut microbiota in the development of NAFLD is still unclear. There are several kinds of n-3 PUFAs: docosahexaenoic acid (DHA, 22:6(n-3)) and eicosapentaenoic acid (EPA, 20:5(n-3)), which are common in deep sea fish oil, and alpha-linolenic acid (ALA, 18:4(n-3)), which is common in particular plant oils, such as perilla oil. The different effects between n-3 ALA and DHA or EPA against NAFLD remain unknown. In the present study, we illustrated that n-3 PUFAs induced intestinal microbiome alteration at least partly contributes to the amelioration of high-fat induced NAFLD, and perilla oil is as potent as fish oil.

## 2. Method

### 2.1. Animals and Diets

Male Sprague-Dawley (S-D) rats (8-9 weeks old weighing 180~200 g) were purchased from Tonji Medical College at Huazhong University of Science and Technology (Wuhan, China). They were housed five per cage and maintained in a 12-hour light/dark cycle under standard laboratory settings with a room temperature of 22°C ± 1°C, relative humidity of 60%  ±  10%, and 20 air changes per hour. After one week acclimation to the lab conditions, the rats were randomly allocated to four groups (10 animals per group), and each group was fed one of the following three diets for 16 weeks: normal chow diet with 10 kcal% fat (NOR group), Western style lard-rich diet with 45 kcal% fat and 2% cholesterol (w/w) (HFD group), fish-oil-rich diet with 10% fish oil (w/w) and a total 45 kcal% fat with 2% cholesterol (w/w) (FOH group), or perilla-oil-rich diet with 5.5% perilla oil (w/w) and a total 45 kcal% fat with 2% cholesterol (w/w) (POH group). The detail information of diets were presented in supplementary Table  1 (see Supplementary Material available online at http://dx.doi.org/10.1155/2016/9462571). The animals were given water and diet ad libitum. This animal study was conducted according to the Guidelines for the Care and Use of Experimental Animals, and the protocol was approved by Laboratory Animal Ethics Committee of Wuhan Polytechnic University (ID number: 20121009006). At the end of 16th weeks feeding, 12 hours fasted rats were sacrificed with CO_2_ suffocate. Blood was then distributed into a heparinized tube (6–8 mL) and centrifuged at 1,000 g for 15 min at 4°C, and plasma was collected and stored at −80°C until analysis. Liver was quickly removed, rinsed with 0.9% sodium chloride solution and weighed; a portion of the right hepatic lobe was either frozen in liquid nitrogen and kept at −80°C, or fixed in 10% neutral buffered formalin and embedded in paraffin for histological studies.

### 2.2. Measurement of Serum Parameters

Serum total cholesterol (TCH), triglyceride (TG), low-density lipoprotein cholesterol (LDL-C), and the activity of serum alanine aminotransferase (ALT) were determined using respective diagnostic kits (Jiancheng Technology, Nanjing, China) according to the manufacturer's instructions. All parameters were measured in duplicate.

### 2.3. Histological Studies

The fixed liver tissues were embedded in paraffin and sectioned at 5 *μ*m thickness, stained with hematoxylin and eosin. The stained slides were observed photomicrographically, and the degree of liver steatosis was examined blindly under a Nikon N80i microscope.

### 2.4. Hepatic RNA Extraction and Quantitative RT-PCR

Total hepatic RNA was isolated from liver tissue using the TRIZOL reagent following supplier's protocol (Takara, Dalian, China). RNA quantity and quality were determined using the Nanodrop ND-1000 spectrophotometer (Thermo Fisher Scientific) at a wavelength of 260/280 nm. cDNA was generated from 5 *μ*g of total RNA using M-MLV reverse transcriptase. A 20 *μ*L amplification reaction consisted of SYBR Green I PCR Master Mix (Takara, Dalian, China) with 300 nM of both reverse and forward primers. All reactions were performed on a 7500 Real-time PCR System (Applied Biosystems). The thermal cycling conditions were 2 min at 50°C, and 3 min at 95°C, followed by 40 repeats at 95°C for 15 s, 60°C for 30 s, and 72°C for 30 s. The fluorescent products were detected at the last step of each cycle in the reactions. To control for variations in the reactions, the amount of target mRNA was normalized to invariable control gene glyceraldehyde-3-phosphate dehydrogenase (Gapdh) expression. The comparative threshold cycle (Ct) method was used to determine the amount of target gene normalized to Gapdh and relative to a calibrator 2^−ΔΔCt^. The purity of PCR products was verified by melting curves and gel electrophoresis.

### 2.5. Genomic DNA Extraction, Sequencing, and Quantitative Analysis of the Microbiome Composition

Fecal samples after collection were immediately kept at −80°C and stored until being analysed. The DNA was extracted from rectal stool samples of NOR, HFD, FOH, and POH rats. QIAmp DNA Stool Mini Kit (Qiagen, Germany) was used for stool sample (200 mg each) DNA extraction according to the manufacturer's instructions. 16S ribosomal DNA (rDNA) sequences were amplified and pyrosequenced on an Illumina MiSeq platform. Pyrosequence reads were analyzed in the Quantitative Insights into Microbial Ecology (QIIME) software version 1.6.0. For taxonomic assignment, sequence reads were grouped into operational taxonomic units (OTUs) at a sequence similarity level of 97%.

### 2.6. Validation of the Quantification of Relevant Bacteria

To verify the relative abundance of* Akkermansia* and* Escherichia* between groups, we performed 16S rRNA gene-targeted RT-PCR as shown previously [22]. Reactions were performed in triplicate. The copy number of target DNA was determined by comparison with serially diluting standards (10^1^ to 10^7^ copies of plasmid DNA containing the respective amplicon for each set of primers). Bacterial quantity was expressed as Log_10_ (bacterial cells per gram of stool).

### 2.7. Statistical Analysis

All data are presented as the mean ± standard error of mean (SEM) with normal distribution. The significance of differences in data between the groups were determined by one-way ANOVA followed by Turkey's test for equality of variances using SPSS 17.0 (IBM, USA). Differences were considered statistically significant at *p* < 0.05.

## 3. Results

### 3.1. Fish Oil and Perilla Oil Ameliorated High-Fat Diet-Induced Hepatic Steatosis

**Figure 1:**
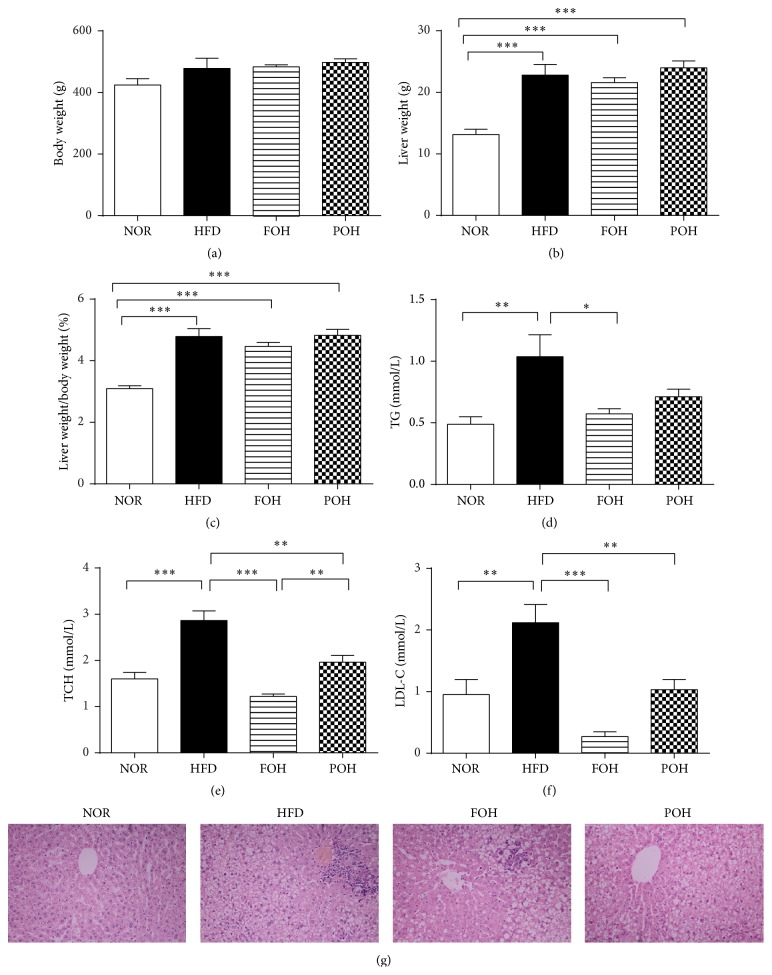
Fish oil and perilla oil consumption reversed HFD-induced hepatic steatosis. Effects of HFD, FOH, and POH on (a) body weight, (b) liver weight, (c) liver/body weight ratio, (d) serum TG, (e) serum TCH, (f) serum LDL-c, and (g) hepatic H&E staining were shown. Data were presented as mean ± SEM, *n* = 6. ^*∗*,*∗∗*,*∗∗∗*^ *p* < 0.05, 0.01, 0.001, respectively.

Male SD rats were fed normal lab chow (NOR), high-fat diet (HFD), and high-fat diet combined with fish oil (FOH) or perilla oil (POH) for 16 weeks. All three high-caloric diets (HFD, FOH, and POH) caused significant increases in liver weight and liver/body weight ratio, but no effects on body weight were observed. However, only HFD led to significantly higher serum TG, TCH, and LDL-c levels compared with NOR group. The consumption of either fish oil or perilla oil reduced HFD-induced hypercholesterolemia, but only fish oil reversed the HFD-induced hypertriglyceridemia. Histological staining presented similar results, both fish oil and perilla oil intake slightly reversed HFD-induced serious hepatic steatosis (Figure 1).

### 3.2. Fish Oil and Perilla Oil Attenuated High-Fat Diet-Induced Hepatic Damage and Inflammation

**Figure 2:**
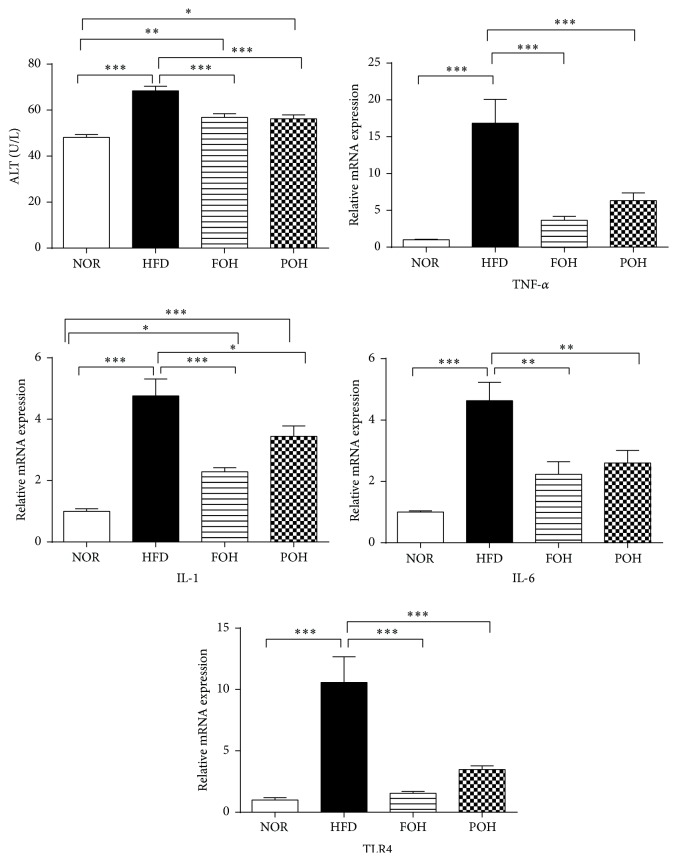
Effects of fish oil and perilla oil on the expression of hepatic proinflammatory genes in HFD-induced NAFLD rats. Data were presented as mean ± SEM, *n* = 6. ^*∗*,*∗∗*,*∗∗∗*^ *p* < 0.05, 0.01, 0.001, respectively.

Excessive fat consumption not only induced lipid ectopic accumulation in liver but also led to liver injury. Serum ALT is one of best characterized markers of liver injury. The ALT values of rats fed HFD, FOH, and POH were all significantly higher than NOR group. The ALT values of rats in FOH and POH groups were both moderately and significantly lower than that of HFD group. Besides the liver injury, HFD induced serious hepatic inflammation. Macrophage infiltration was observed in FOH and POH groups via H&E staining. The infiltrated macrophages were slightly less than HFD group. Hepatic proinflammatory cytokines analysis showed coincident results. The hepatic mRNA expression levels of TNF-*α*, IL-1*β*, and IL-6 were all significantly increased in HFD groups compared with that of control NOR group. In FOH and POH groups, the expression levels of these proinflammatory cytokines were all significantly lower than HFD group; only the expression level of IL-1*β* in FOH and POH groups was significantly higher than that of NOR group. Toll-like receptor 4 (TLR4), the key pattern-recognition receptor of LPS, plays a crucial role in innate immune system and links to metabolic syndrome [23]. Both fish oil and perilla oil consumption completely reversed HFD-induced high expression of TLR4, to a level that is similar to NOR group (Figure 2).

### 3.3. Diet-Induced Changes in Intestinal Microbiome

**Figure 3:**
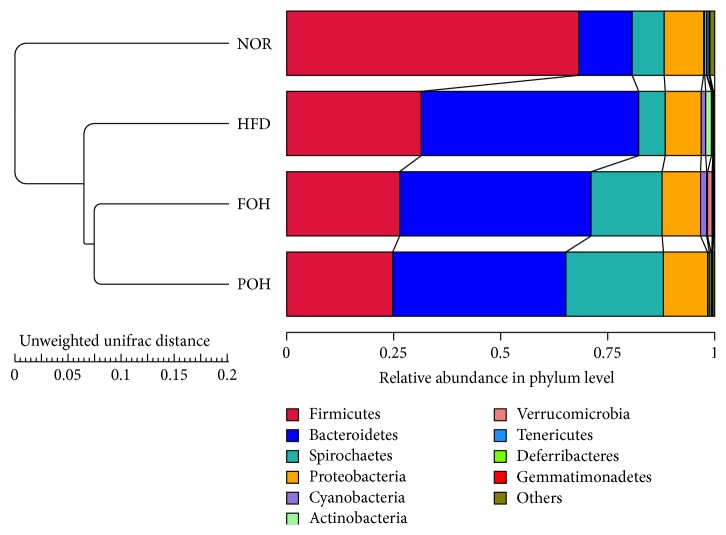
Unweighted Pair Group Method with Arithmetic mean (UPGMA) clustering clustering of microbiota composition for each treatment group.

Gut microbiota affects the digestion and absorption of diets. Meanwhile, the daily diets regulate the quantity and variety of microbiota. Earlier studies have demonstrated that feeding of western style diet with high saturated fat leads to the change of gut microbiota [21]. In the present study, we examined the effects of HFD and HFD combined with fish oil or perilla oil on gut microbiota by pyrosequencing-based analysis of bacterial 16S ribosomal RNA (V4 region) in faeces. Unweighted Pair Group Method with Arithmetic mean (UPGMA) clustering illustrated that rats fed high-caloric diets HFD, FOH, and POH were grouped closely, and the rats fed NOR were branched separately, which suggests high fat consumption remarkably altered the structure of the gut microbiota. Furthermore, rats fed FOH and POH were grouped more closely compared with HFD, which suggests that n-3 PUFAs intake presented similar effects on gut microbiota community (Figure 3).

#### 3.3.1. Comparison of Microbiomes at the Phylum Level

**Figure 4:**
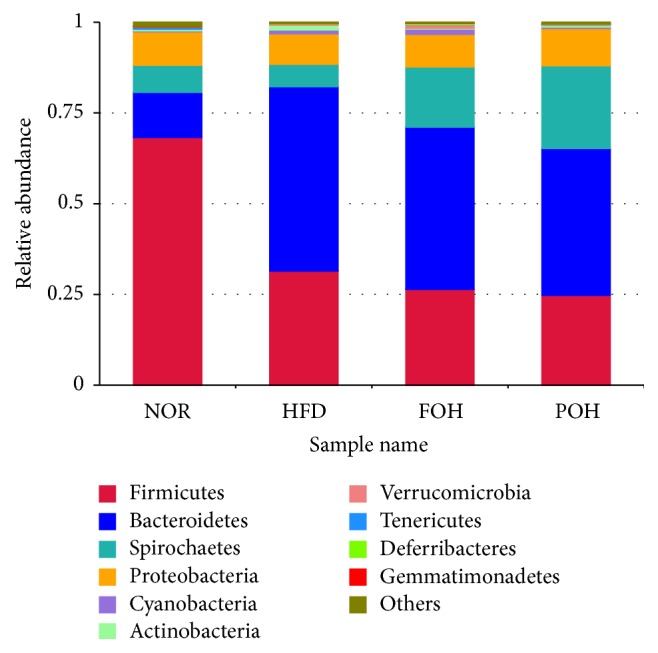
The relative abundance of gut microbiota at the phylum level.

Ten major bacteria phyla were detected in gut microbiomes in this study (Figure 4). Bacteroides and Firmicutes were the dominant phyla in samples. Taxonomic profiling demonstrated that a dramatic increase in Bacteroides and a decrease in Firmicutes were observed in three high-caloric feeding groups, comparing with the low-fat NOR group. The ratio of Firmicutes to Bacteroidetes in NOR group was 5.53, while in HFD, FOH, and POH group it was 0.62, 0.59, and 0.61, respectively. Spirochaetes, Proteobacteria, Cyanobacteria, Actinobacteria, and Verrucomicrobia are other several phyla that exhibited >1% relative abundance in at least one of the groups. Among them, both fish oil and perilla oil drastically increased the abundance of Spirochaetes to 16.59% and 22.77%, respectively. The relative abundance of Proteobacteria in HFD group was slightly lower than other three groups. Only HFD group had a higher abundance of Actinobacteria, and FOH was the only group with a higher abundance of Verrucomicrobia.

#### 3.3.2. Comparison of Microbiomes at the Family and Genus Levels

Consistent with the high levels of Firmicutes and Bacteroidetes, the dominant bacteria detected at the family level included* Lachnospiraceae, Prevotellaceae, Ruminococcaceae, Bacteroidaceae, S24-7*, and* Peptostreptococcaceae* (Table 1). Within phylum Firmicutes, three high-caloric diets decreased the populations of* Lachnospiraceae and Ruminococcaceae* but increased the population of* Peptostreptococcaceae.* Within phylum Bacteroidetes, high-caloric diets increased the population of* Prevotellaceae, Bacteroidaceae,* and* S24-7*, which contribute to the increased abundance of Bacteroidetes. Moreover, the relative abundance of* Prevotellaceae* in FOH and POH groups was lower than HFD group.

At Genus level, within phylum Firmicutes, all three high-caloric diets induced a lower relative abundance of* Ruminococcus, Oscillospira, and Clostridium.* Particularly, though the population of family* Lachnospiraceae* was much lower in three high-caloric diets feeding groups than NOR group, the relative population of genus* Roseburia* was higher in these groups, and only HFD feeding increased the population of* Blautia.* Similarly, within family* Ruminococcaceae*, only HFD elevated the population of* Faecalibacterium,* compared with other three groups; and in family* Lactobacillaceae*, the population of the only abundant Genus* Lactobacillus* in HFD group was higher than other three groups.

Within phylum Bacteroidetes, three high-caloric groups induced a higher abundance of* Prevotella* and* Bacteroides* at genus level. As* Prevotella* is the only detected genus in family* Prevotellaceae,* the abundance of* Prevotella* in FOH and POH group was slightly lower than that in HFD group. Moreover, HFD feeding raised the relative abundance of genus* Escherichia* and* Sutterella* in phylum Proteobacteria and genus* Bifidobacterium* in phylum Actinobacteria compared with other three groups.

**Figure 5:**
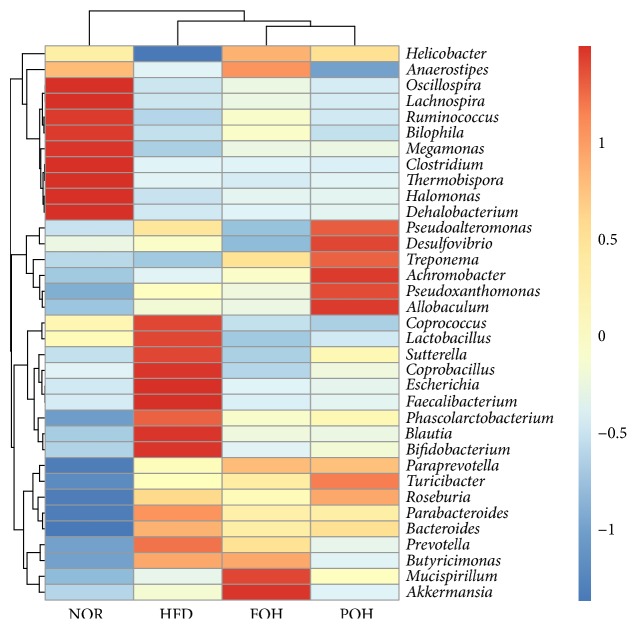
Hotmap of identifed germs with change between each two groups.

Interestingly, only high fish oil diet feeding increased the abundance of genus* Akkermansia,* but another n-3 PUFA enriched diet, POH diet, did not. In order to verify this result, we examined the relative abundance of* Akkermansia* 16S rRNA by real-time PCR, and the result was consistent with the pyrosequencing data; the relative abundance of* Akkermansia* in FOH group was significantly higher than other three groups (Figure 5).

#### 3.3.3. Validation of Intestinal Microbiota Quantification

**Figure 6:**
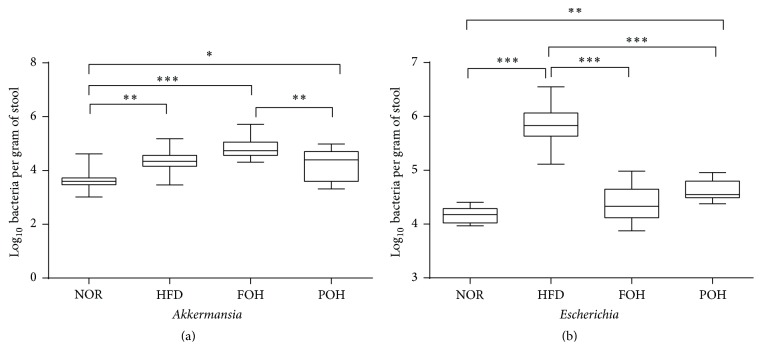
Relative abundance of* Akkermansia* and* Escherichia* in faecal samples. Data were presented as mean ± SEM, *n* = 6. ^*∗*,*∗∗*,*∗∗∗*^ *p* < 0.05, 0.01, 0.001, respectively.

To quantify the 16S RNA sequencing data of intestinal microbiome, qPCR was performed to measure the relative abundance of* Akkermansia* and* Escherichia* in each group. The PCR results were consistent with the gut microbiota 16S rRNA sequencing results, the relative abundance of* Akkermansia* in FOH group was significantly higher than NOR and HFD groups; high saturated fat feeding increased the relative abundance of* Escherichia* significantly, but high fish oil feeding did not (Figure 6). The qPCR results confirmed that the 16S sequencing data were reliable.

## 4. Discussion

NAFLD, a hepatic feature of metabolic syndrome, was mainly caused by unbalanced caloric intake and expenditure, particularly excess fat consumption. The excessive fatty acids uptake and* de novo* lipogenesis in liver lead to lipid ectopic accumulation in hepatocyte attract macrophages infiltration and proinflammatory cytokines release and then develop to hepatic steatosis with inflammation and fibrosis, known as NASH [24]. Nowadays, gut microbiota attracts more and more attentions for their important roles in the development of NAFLD. Bacteria in intestine take in charge of producing of short-chain fatty acids such as acetate, propionate, and butyrate by fermenting the nondigestible carbohydrates. In this way, gut microbiota contributes to intestine health, including but not limited to maintain the intestinal permeability [8].

As daily diets play the central role in the development of NAFLD, nutrition intervention is potential to ameliorate the development of NAFLD; particularly, n-3 PUFAs attract lots of attentions for their anti-inflammatory and antioxidative effects. Animal experiments have illustrated the protective effects of n-3 PUFAs against NAFLD such as reducing hepatic steatosis and improving insulin sensitivity [25]. Clinical studies confirmed the experimental outputs and showed that n-3 PUFAs consumption could reduce both hepatic fat and aspartate aminotransferase levels [26]. Our previous study also demonstrated the protective effects of n-3 PUFAs enriched fish oil against diet-induced NAFLD [27]. The effects of fish oil against NAFLD have been studied widely; however, the effects of perilla oil on NAFLD remain unclear. In the present study, we compared the different effects of fish oil and perilla oil against diet-induced NAFLD; furthermore, we studied the effects of these two oils on gut microbiota. As we known, this is the first study to compare the different effects of fish oil and perilla oil against diet-induced NAFLD, particularly the modification of gut microbiota composition.

After 16 weeks of feeding, the rats in three high caloric diet groups, HFD, FOH, and POH, have similar bodyweight and liver weight; however, only HFD feeding induced significantly higher serum TG, TCH, and LDL-c compared with low-fat control group, both FOH and POH groups showed significantly lower serum TCH and LDL-c levels, but only fish oil consumption reduced serum TG significantly, and FOH has a lower TCH level than POH, suggests fish oil has better protective effects against HFD-induced hyperlipemia than perilla oil. Our previous study has shown that n-3 PUFAs may ameliorate serum and liver fatty acids and cholesterol transport and metabolism via regulating hepatic metabolic gene expression such as SREBP-1 and circadian clock-related genes such as* per2* and* per3* [27]. On the other hand, daily diets intake alter the components of gut microbiota, as well as gut microbiota alter the digestion and absorption of diets, then affect the metabolism, particularly carbohydrate, lipids, and cholesterol [8]. Many studies have indicated gut microbiota play an important role in fatty acid and cholesterol metabolism [28]. As the intestinal microbiome of FOH and POH was grouped more closely than HFD group (Figure 3), which suggests the effects of fish oil and perilla oil on HFD-induced hyperlipemia may partly contribute to the change of gut microbiota.

Lipid ectopic accumulation in liver attracts the infiltration of inflammatory cells, particularly macrophages, and then results in hepatic high level proinflammatory cytokines, induces chronic low-grade inflammation in liver, known as metaflammation [29]. In the present study, HFD feeding induced significant higher level of proinflammatory cytokines in liver, including IL-1*β*, TNF-*α*, and IL-6. The anti-inflammatory effects of n-3 PUFAs are believed to contribute to the protective effects against the development of NAFLD [19, 30]. Our preliminary data confirmed the anti-inflammatory effects of fish oil against HFD-induced hepatic metaflammation [27], and in the current study we found both fish oil and perilla oil present the effects to reduce the hepatic level of proinflammatory cytokines, the levels of IL-1*β*, TNF-*α*, and IL-6 in FOH and POH groups were both significantly lower than HFD group (Figure 2).

Gut microbiota plays a critical role in the development of NAFLD, unhealthy high caloric diets induced alteration of intestinal microbiota is believed a major reason of systemic metaflammation [31]. High-fat diet feeding induces higher relative abundance of Gram-negative bacteria in gut and then leads to elevated level of LPS in portal blood and liver [32]. In the current study, thought the ratio of Firmicutes-to-Bacteroidetes was similar between HFD, FOH, and POH group, the percentage of total Gram-negative bacteria in fish oil and perilla oil feeding groups were lower than HFD feeding group. TLR4 is the key pattern-recognition receptor of LPS, the hepatic expression of TLR4 in POH group and FOH group was both significantly lower than HFD group (Figure 2), indicates n-3 PUFAs consumption reduced hepatic LPS level, which is coincident with the microbiome data. Particularly, the Gram-negative* Prevotella* was much higher in HFD group compared with low-fat control diet feeding animals (17.28% versus 2.74%), but in the gut of fish oil and perilla oil fed rats, the relative abundance of* Prevotella* were lower than HFD group (12.98% and 7.89%, resp.).* Prevotella* was thought to take part in the development of NASH [33]. Moreover, Choi and the colleagues found that DHA, a n-3 PUFA, depresses* Prevotella* intermedia LPS-induced inflammation in macrophage [34], suggesting that n-3 PUFAs may ameliorate HFD-induced hepatic metaflammation via both reducing the abundance of Gram-negative germs in intestinal and suppressing Gram-negative germs induced inflammation in liver.

Besides the LPS, endogenous ethanol is believed to be involved in the development of inflammation in NAFLD and NASH patients [33, 35]. In the current study, we found the relative abundance of alcohol-producing* Escherichia* was significantly higher in HFD group than NOR group, but in FOH group, the relative abundance of* Escherichia* was similar as NOR group (Figure 6). The low level of* Escherichia* in n-3 PUFAs fed animals may contribute to the decreased metaflammation in liver.

Many studies have demonstrated the bacterium* Akkermansia* can protect against HFD induced metabolic disorder and metaflammation via inducing endocannabinoids synthesis and gut peptides expression [18, 36]. Unexpected, in the present study, the relative abundance of* Akkermansia* was low in NOR group, and HFD feeding slightly increased the percentage of* Akkermansia*. Notably, fish oil consumption significantly elevated the relative abundance of* Akkermansia* in gut, but perilla oil not (Figure 6). Why only fish oil promotes the relative abundance of* Akkermansia* is worth further study.

Patterson and the colleagues have demonstrated the effects of flaxseed/fish oil mixture on mouse intestinal microbiome [37]. As far as we known, the present study was the first one to compare the different effects of ALA enriched perilla oil and DHA/EPA enriched fish oil on HFD-induced metabolic disorder and gut dysbiosis, and our findings will help to understand the nutritional benefits of perilla oil on health.

## Supplementary Material

Supplementary Table 1. Nutrient composition, energy ratio and fatty acids profile of experimental diets. The detail information of animal diets was presented. The fatty acids profile of diets was measured by GC-MS.

## Figures and Tables

**Table 1:** Relative abundance of identified germs.

	NOR	(%)		HFD	(%)		FOH	(%)		POH	(%)	
Bacteroidetes	**12.36 **			**50.83 **			**44.67 **			**40.46 **		
Prevotellaceae		*2.74 *			*17.28 *			*12.98 *			*7.89 *	
*Prevotella*			2.74			17.28			12.98			7.89
Bacteroidaceae		1.07			9.54			8.47			9.77	
*Bacteroides*			0.98			5.77			4.67			5.10
S24-7		5.69			11.79			10.61			11.25	
Rikenellaceae		0.29			0.41			0.63			0.42	
Porphyromonadaceae		0.23			1.84			1.32			1.36	
Parabacteroides			0.17			1.83			1.32			1.35
Firmicutes	**68.40 **			**31.43 **			**26.47 **			**24.72 **		
Lachnospiraceae		*35.27 *			*9.27 *			*7.94 *			*6.49 *	
*Blautia*			0.07			0.73			0.24			0.22
*Roseburia*			0.21			1.19			0.96			1.33
*Lachnospira*			0.15			0.01			0.03			0.02
*Coprococcus*			0.13			0.22			0.07			0.06
*Anaerostipes*			0.11			0.09			0.12			0.07
Ruminococcaceae		*19.21 *			*8.34 *			*7.97 *			*5.96 *	
*Ruminococcus*			1.61			0.48			0.82			0.57
Oscillospira			6.84			1.70			2.47			1.85
*Faecalibacterium*			0.28			3.45			0.32			0.47
Veillonellaceae		*0.37 *			*1.56 *			*0.84 *			*0.93 *	
Clostridiaceae		1.13			0.23			0.16			0.12	
*Clostridium*			0.85			0.04			0.04			0.03
Lactobacillaceae		*0.55 *			*0.76 *			*0.37 *			*0.47 *	
*Lactobacillus*			0.48			0.70			0.33			0.38
Veillonellaceae		*0.37 *			*1.56 *			*0.84 *			*0.93 *	
Peptostreptococcaceae		1.40			7.14			5.99			6.43	
Thermoanaerobacteraceae		0.84			0.05			0.02			0.02	
Spirochaetes	**7.47 **			**6.21 **			**16.59 **			**22.77 **		
Spirochaetaceae		*7.47 *			*6.21 *			*16.59 *			*22.77 *	
*Treponema*			7.47			6.21			16.59			22.77
Proteobacteria	**9.20 **			**8.38 **			**8.93 **			**10.36 **		
Enterobacteriaceae		*0.07 *			*0.72 *			*0.08 *			*0.11 *	
*Escherichia*			0.06			0.72			0.08			0.11
Campylobacteraceae		0.05			0.01			0.01			0.01	
*Campylobacter*			0.05			0.01			0.01			0.01
Alcaligenaceae		*0.39 *			*1.13 *			*0.35 *			*0.75 *	
*Sutterella*			0.37			1.11			0.32			0.65
*Helicobacteraceae*		*2.91 *			*0.89 *			*3.48 *			*3.15 *	
*Helicobacter*			2.89			0.89			3.48			3.15
Desulfovibrionaceae		1.08			1.36			0.91			1.39	
*Bilophila*			0.19			0.03			0.07			0.03
*Desulfovibrio*			0.87			0.93			0.69			1.29
Alcaligenaceae		0.39			1.13			0.35			0.75	
Cyanobacteria	**0.31 **			**1.06 **			**1.45 **			**0.43 **		
Actinobacteria	**0.45 **			**1.36 **			**0.21 **			**0.40 **		
Bifidobacteriaceae		*0.01 *			*1.27 *			*0.18 *			*0.33 *	
*Bifidobacterium*			0.01			1.27			0.18			0.33
Verrucomicrobia	**0.04 **			**0.31 **			**1.05 **			**0.18 **		
Verrucomicrobiaceae		*0.03 *			*0.31 *			*1.04 *			*0.16 *	
*Akkermansia*			0.02			0.28			1.01			0.14
