# Supplementary material for: Perilla Oil Has Similar Protective Effects of Fish Oil on High-Fat Diet-Induced Nonalcoholic Fatty Liver Disease and Gut Dysbiosis
Source: Biomed Res Int. 2016 Mar 9;2016:9462571. doi: 10.1155/2016/9462571 (PMC4804047; doi:10.1155/2016/9462571)
Supplement: Supplementary file 1 — Supplementary Table 1. Nutrient composition, energy ratio and fatty acids profile of experimental diets. The detail information of animal diets was presented. The fatty acids profile of diets was measured by GC-MS. [file 9462571.f1.docx]

**Supplementary table 1. Nutrient composition, energy ratio and fatty acids profile of experimental diets.**

|  | 10% fat diet | 45% fat diets | | |
| --- | --- | --- | --- | --- |
|  | **NOR** | **HFD diet** | **FOH diet** | **POH diet** |
| Total energy, Kcal/100g | 386 | 476 | 476 | 476 |
| Protein contributed energy, % | 20 | 12 | 12 | 12 |
| Carbohydrate contributed energy, % | 70 | 43 | 43 | 43 |
| Fat contributed energy, % | 10 | 45 | 45 | 45 |
| Basal chow, g | 100 | 78 | 78 | 78 |
| Lard, g | / | 20 | 10 | 14.9 |
| Fish oil, g | / | / | 10 | / |
| Perilla seeds oil | / | / | / | 5.1 |
| Cholesterol, g | / | 2 | 2 | 2 |
|  |  |  |  |  |
| **Fatty acids profile (%of total fatty acid)** |  |  |  |  |
| C14:0 | 0.16 | 1.26 | 4.09 | 0.95 |
| C16:0 | 15.56 | 25.11 | 23.12 | 20.78 |
| C18:0 | 29.2 | 20.06 | 12.54 | 16.39 |
| **∑ Saturated FAs** | **44.91** | **46.43** | **39.75** | **38.12** |
| C16:1 n-7 | 1.09 | 1.86 | 5.18 | 1.38 |
| C18:1 n-9 | 10.03 | 32.47 | 27.15 | 28.1 |
| **∑ Monounsaturated FAs** | **11.12** | **34.34** | **32.33** | **29.48** |
| C16:3 n-3 | nd | nd | 0.64 | nd |
| C16:4 n-3 | 2.31 | 0.33 | 1.21 | 0.33 |
| C18:3 n-3 | 3.61 | 1.08 | 0.75 | 13.72 |
| C18:4 n-3 | tr | nd | 1.37 | nd |
| C20:5 n-3 | nd | tr | 8.11 | nd |
| C22:5 n-3 | nd | nd | 0.94 | nd |
| C22:6 n-3 | nd | nd | 5.75 | nd |
| **∑ n-3 Polyunsaturated FAs** | **5.92** | **1.41** | **18.78** | **14.05** |
| C18:2 n-6 | 38.02 | 17.67 | 8.6 | 18.24 |
| C20:4 n-6 | tr | 0.15 | 0.54 | 0.11 |
| **∑ n-6 Polyunsaturated FAs** | **38.02** | **17.82** | **9.14** | **18.35** |

Some fatty acids, of which the contents are minor, trace amountor not detected, such as C22:0, C24:0, C14:1, C20:2n−6, and C20:3n−6, were not listed in the table. tr: trace; nd: non-detected.
